# The Effects of *Aronia melanocarpa* ‘Viking’ Extracts in Attenuating RANKL-Induced Osteoclastic Differentiation by Inhibiting ROS Generation and c-FOS/NFATc1 Signaling

**DOI:** 10.3390/molecules23030615

**Published:** 2018-03-08

**Authors:** Mithun Ghosh, In Sook Kim, Young Min Lee, Seong Min Hong, Taek Hwan Lee, Ji Hong Lim, Trishna Debnath, Beong Ou Lim

**Affiliations:** 1 Department of Applied Life Science, College of Biomedical & Health Science, Konkuk University, Chungju-si, Chungcheongbuk-do 27478, Korea; mithunghoshmg@gmail.com (M.G.); inskim@sookmyung.ac.kr (I.S.K.); waz12345@naver.com (Y.M.L.); hongsm0107@kku.ac.kr (S.M.H.); thlee@ag-health.com (T.H.L.); jhlim@kku.ac.kr (J.H.L.); 2 Ahn-Gook Health, LTD, Seoul 07445, Korea; 3 Department of Food Science and Biotechnology, Dongguk University-Seoul, Goyang-si, Gyeonggi-do 10326, Korea; trishna_rahul@yahoo.com

**Keywords:** osteoclast, RANKL, phenol, ROS, free radical

## Abstract

This study aimed to determine the anti-osteoclastogenic effects of extracts from *Aronia melanocarpa* ‘Viking’ (AM) and identify the underlying mechanisms in vitro. Reactive oxygen species (ROS) are signal mediators in osteoclast differentiation. AM extracts inhibited ROS production in RAW 264.7 cells in a dose-dependent manner and exhibited strong radical scavenging activity. The extracts also attenuated the number of tartrate-resistant acid phosphatase (TRAP)-positive multinucleated osteoclasts. To attain molecular insights, the effect of the extracts on the signaling pathways induced by receptor activator of nuclear factor kappa B ligand (RANKL) were also investigated. RANKL triggers many transcription factors through the activation of mitogen-activated protein kinase (MAPK) and ROS, leading to the induction of osteoclast-specific genes. The extracts significantly suppressed RANKL-induced activation of MAPKs, such as extracellular signal-regulated kinase (ERK), c-Jun-*N*-terminal kinase (JNK) and p38 and consequently led to the downregulation of c-Fos and nuclear factor of activated T cells 1 (NFATc1) protein expression which ultimately suppress the activation of the osteoclast-specific genes, cathepsin K, TRAP, calcitonin receptor and integrin β_3_. In conclusion, our findings suggest that AM extracts inhibited RANKL-induced osteoclast differentiation by downregulating ROS generation and inactivating JNK/ERK/p38, nuclear factor kappa B (NF-κB)-mediated c-Fos and NFATc1 signaling pathway.

## 1. Introduction

The human bone is a rigid organ that achieves homeostasis through a balance between bone formation and resorption. The formation of bone depends on osteoblastic proliferation, alkaline phosphatase activity, collagen synthesis and mineralization, whereas osteoclast formation and tartrate-resistant acid phosphatase (TRAP) activity are closely associated with bone resorption [1]. The bone mass of adults is determined by two critical activities: osteoclast-mediated bone resorption and osteoblast-induced bone formation. Numerous bone-associated disorders, such as osteoporosis, osteomalacia and osteopetrosis, result from a disruption of this balance [2,3,4]. Osteoclasts are multinucleated giant cells derived from a hematopoietic stem cell monocyte/macrophage lineage. Macrophage colony-stimulating factor (M-CSF) and (RANKL) are the two key cytokines that regulate the formation of osteoclasts from monocyte/macrophage lineage precursor cells through a differentiation process [4]. RANKL delivers important signals for osteoclast differentiation, whereas osteoclasts and their precursor cells receive survival signals from M-CSF [5]. The activation of three major mitogen-activated protein kinases, ERK, JNK and p38, are induced by RANKL signaling in osteoclast precursor cells. Subsequently, MAPKs initiate the induction and activation of many transcription factors associated with the expression of osteoclast-specific genes—namely, NFATc1, activator protein 1 (AP-1) and NF-κB [6]. Activated ERK and JNK can directly phosphorylate c-Fos and c-Jun, respectively. Therefore, transcription factor AP-1, which is a heterodimer of proteins from the Fos and Jun families, could be a target of ERK and JNK in RANKL-induced osteoclast precursor cells. A previous study reported that c-Fos and NFATc1 expression was induced by RANKL in osteoclast precursor cells [7]. NF-κB is also considered a crucial transcription factor for osteoclast differentiation induced by RANKL. In response to RANKL signaling, the proteasome-mediated degradation of inhibitory kappa B (IκB) characterized the activation and nuclear translocation of NF-κB in osteoclast precursor cells [8]. Iotsova et al. observed a noticeable decline in the number of osteoclasts in mice with a double knockout of p50 and p52 [9].

Many plants and their derivatives have been used in folk medicine for the treatment of osteoporosis. Recently, various natural compounds have been reported to possess inhibitory effects on osteoclast differentiation. Scoparone, a bioactive component of *Artemisia capillaris* Thunb., sauchinone, a lignin from *Saururus chinensis*, bavachalcone from *Psoralea corylifolia* and berberine, present in *Coptis chinensis*, have all been previously reported to possess anti-osteoclastic effects. 

*Aronia melanocarpa* ‘Viking’ (AM), a member of the Rosaceae family, exhibits noticeable antioxidant potential. AM contains numerous polyphenolic compounds, including anthocyanins (cyanidin 3-*O*-galactoside, cyanidin 3-*O*-arabinoside, cyanidin 3-*O*-xyloside and cyaniding 3-*O*-glucoside), phenolic acids (caffeic acid, ferulic acid and chlorogenic acid), flavonoids (quercetin 3-*O*-vicianoside, quercetin 3-*O*-robinobioside and other quercetin glucosides) [10]. Our study conducted the determination of anthocyanin compounds in AM extracts by high performance liquid chromatography (HPLC) and confirmed the presence of four important anthocyanin compounds. In this study, we investigated the effects of the water (AM-DW) and ethanol (AM-EtOH) extracts of AM on RANKL-induced osteoclastic differentiation. Our findings demonstrated that the AM extracts suppressed RANKL-induced osteoclastic differentiation through inactivation of MAP kinase pathway which consequently downregulate the expression of transcription factors such as NF-κB, c-Fos and NFATc1.

## 2. Results

### 2.1. Quantitative HPLC Analysis of Anthocyanin Compounds from Aronia melanocarpa ‘Viking’ Extracts

**Figure 1:**
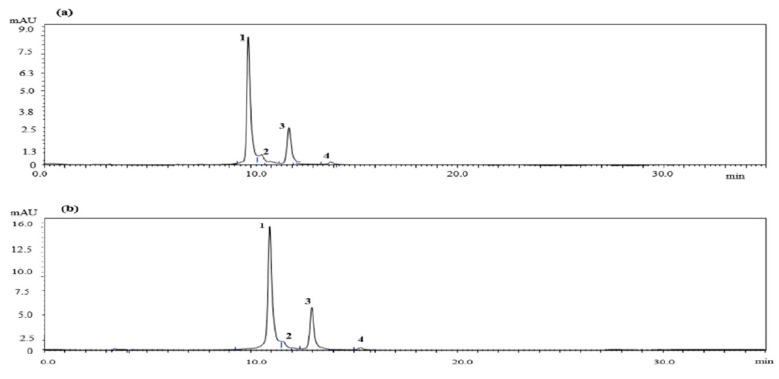
HPLC chromatograms of AM extracts; (**a**) AM-DW; (**b**) AM-EtOH. Peaks: (1) Cyanidin-3-galactoside; (2) Cyanidin-3-glucoside; (3) Cyanidin-3-arabinoside; (4) Cyanidin-3-xyloside.

In this study, four anthocyanin compounds were identified from AM extracts (Figure 1) such as, cyanidin-3-galactoside, cyanidin-3-glucoside, cyanidin-3-arabinoside and cyanidin-3-xyloside. The amounts of the identified compounds are illustrated in Table 1; cyanidin-3-galactoside was more abundant than the other compounds.

### 2.2. Total Phenolic and Flavonoid Contents and Yields of Aronia melanocarpa ‘Viking’ Extracts

To obtain the extracts at acceptable yields and with strong antioxidant activity, the extraction methods are crucial [11]. In this study, the yields were 62.85% and 64.3% for the water and ethanol AM extracts, respectively. Phenolic compounds are the most ubiquitous groups of plant secondary metabolites and known for their antioxidant activities [12]. The chain reaction of lipid peroxidation can be stopped through the donation of a hydrogen from phenolic compounds to a free radical, which acts as an antioxidant. The total amount of the phenolic compounds was evaluated by using the regression equation of the calibration curve and expressed in gallic acid equivalents (GAE). The obtained values were 2617.25 mg and 4004.46 mg GAE per 100 g of dry mass for the water and ethanol extracts of AM, respectively (Table 2). Flavonoids, a diverse group of phytonutrients found in all fruits and vegetables, are recognized as potent antioxidant with considerable effects on human nutrition and health, which are exerted through scavenging and chelating activity [13]. The flavonoid contents, calculated from a calibration curve and expressed in catechin equivalents (CE), were 2004.99 mg and 3896 mg CE per 100 g of dry mass for the water and ethanol extracts of AM, respectively (Table 2).

### 2.3. DPPH Radical Scavenging Activity of Aronia melanocarpa ‘Viking’ Extracts

**Figure 2:**
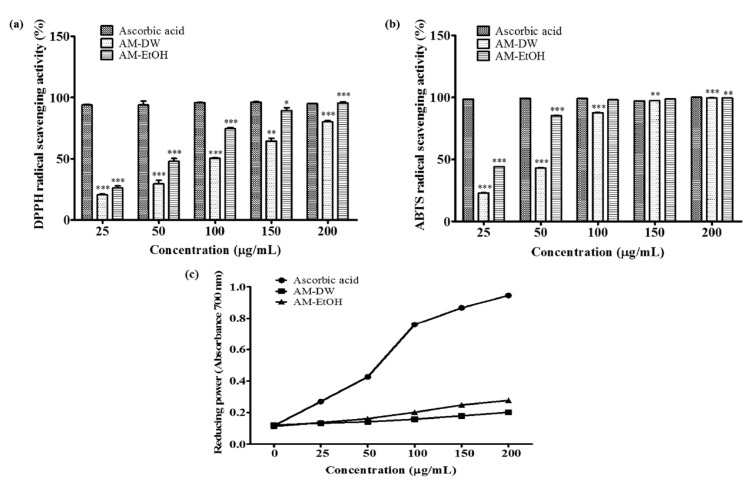
Antioxidant activity of AM extracts. (**a**) DPPH radical scavenging activity of AM extracts; (**b**) ABTS radical scavenging activity of AM extracts. The values are the mean ± S.D. from three independent experiments * *p* < 0.05, ** *p* < 0.01, *** *p* < 0.001 vs. control group. Ascorbic acid used as control; (**c**) Reducing power activity of *Aronia melanocarpa* ‘Viking’ extracts. The data are express as median ± standard deviation (*n* = 3).

1,1-Diphenyl-2-picrylhydrazyl (DPPH) is widely used to measure the free radical scavenging activities of antioxidants. In this assay, antioxidants reduce the stable DPPH radical (purple in color) to the non-radical form DPPH-H (yellow in color) [14]. The DPPH radical scavenging capacities result from the hydrogen donating abilities of the phenolic compounds present in the extracts. The scavenging capacities were 20.58–80.12% and 26.12–95.59% for the water and ethanol extracts of AM, respectively and the concentration ranges were between 25 and 200 μg/mL (Figure 2a). The half-maximal inhibitory concentration (IC_50_) was 98.71 μg/mL for the water extract and 53.45 μg/mL for the ethanol extract; the ethanolic extract of AM was clearly more potent than the water extract of AM.

### 2.4. ABTS Radical Scavenging Activity of Aronia melanocarpa ‘Viking’ Extracts

2,2-Azino-bis (3-ethylbenzo thiazoline-6-sulfonic acid) diammonium salt (ABTS) is a “synthetic radical” that is widely used to measure antioxidant capacity. Phenolic compounds can donate electrons to ABTS radicals. All extracts showed effective radical cation scavenging activity with ABTS radical scavenging activity in the ranges from 23.04–99.585% for the water extract of AM and 44.26–99.42% for the ethanol extract of AM, with IC_50_ values of 54.48 μg/mL and 28.49 μg/mL, respectively (Figure 2b). The IC_50_ values indicated that the ethanolic extract of AM produced more effective inhibition of the ABTS radical. This may be a result of the higher phenolic compound content of the ethanol extract of AM.

### 2.5. Determination of Reducing Power Activity of Aronia melanocarpa ‘Viking’ Extracts

In the reducing power assay, the Fe^3+^/ferricyanide complex is reduced to its ferrous form by the antioxidants. In the ferric to ferrous reduction assay, the electron donation capacity provides an indication of the reductive power; the increased absorbance denotes a higher reduction power. This can be determined spectrophotometrically at 700 nm [15,16]. All the extracts showed mild electron donation activity compared with ascorbic acid. Therefore, the expected capacities of the extracts were inferior to the ascorbic acid. However, the absorbance increased dose-dependently for all the extracts (Figure 2c). The ethanol extract of AM was found to have a more potent effect than the water extract. The absorbance increased to 0.27 for AM ethanol extract and 0.20 for AM water extract for an extract concentration of 200 μg/mL.

### 2.6. Effects of Aronia melanocarpa ‘Viking’ Extracts on Intracellular ROS Generation

**Figure 3:**
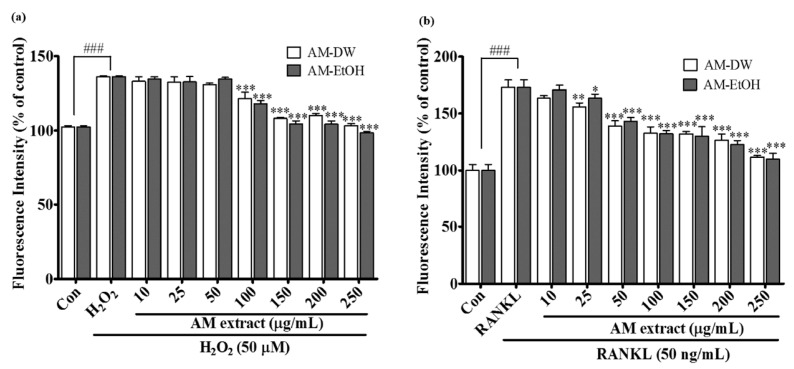
Effects of AM extracts on ROS inhibition. (**a**) Inhibition of H_2_O_2_ induced ROS generation in RAW 264.7 cells by AM extracts; (**b**) Inhibition of RANKL induced ROS generation in RAW 264.7 cells by AM extracts. After 24 h of sample treatment 10 μM of H_2_DCF-DA was added to the cell and incubated for 40 min, following addition of 50 μM H_2_O_2_ or, 50 ng/mL RANKL for 1 h. All data are presented as the mean ± SD of three independent experiments performed with *n* = 3. Compared positive control (not treated group) vs. negative control (only H_2_O_2_ or RANKL treated group ^###^ *p* < 0.001) and negative control vs. sample treated group (* *p* < 0.05, ** *p* < 0.01, *** *p <* 0.001).

Hydrogen peroxide (H_2_O_2_) is an important indicator of ROS accumulation and is used in many studies to induce ROS. Treatment with H_2_O_2_ increased ROS generation by up to 140% but in the presence of the AM extracts ROS generation was significantly reduced as the dose-dependent manner (Figure 3a). The sustained generation of intracellular ROS is considered an important step for RANKL-induced osteoclastogenesis [17]. In various receptor signaling pathways, ROS acts as a second messenger [18]. It is reported that the production of ROS in response to RANKL is increased and acts as an intracellular mediator for the ERK signaling pathway in osteoclast differentiation and activation [17]. Therefore, it was determined if the extracts affected RANKL-induced ROS generation. The intracellular ROS production in RAW 264.7 cells increased 180% after the treatment with RANKL compared with the control group (Figure 3b). The generation of intracellular ROS was dose-dependently attenuated by the treatment of AM extracts within the concentration range from 10–250 μg/mL. Cell viability was investigated in the presence and absence of RANKL to ensure that the treated concentrations were not cytotoxic (Data not shown here). 

### 2.7. Aronia melanocarpa ‘Viking’ Extracts Inhibit RANKL Induced Osteoclastic Differentiation 

**Figure 4:**
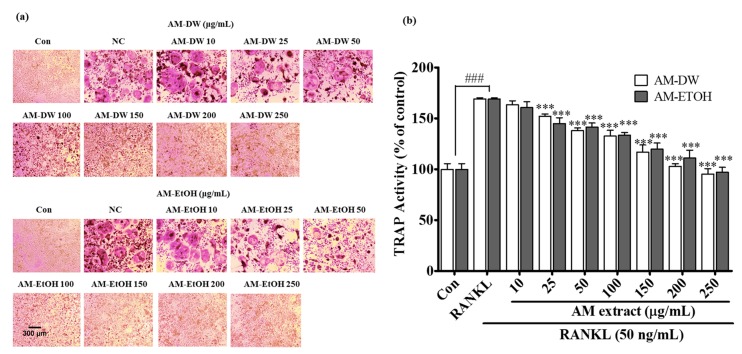
Inhibitory properties of AM extracts on differentiation and TRAP activity (**a**) Inhibitory effects of AM extracts on RANKL-induced round-shaped osteoclast formation originated from RAW 264.7 cells. Cells were exposed to RANKL (50 ng/mL) in the presence and absence of AM extracts for 5 days. The cells were fixed and stained using a leukocyte acid phosphatase (TRAP) kit. Con; positive control, (which was not treated), RANKL; negative control, (which was treated with only RANKL), Sample treated group; RANKL+ sample. TRAP positive multinucleated osteoclasts were visualized in 40× magnification under light microphotography; (**b**) Inhibitory effects on TRAP activity of RANKL induced osteoclast in RAW 264.7 cells by AM extracts. Cells were exposed to RANKL (50 ng/mL) in the presence and absence of AM extracts for 3 days. TRAP activity was measured by TRAP solution assay. All data are presented as the mean ± SD of three independent experiments performed with *n* = 3. Statistical analysis were done by Comparing, Con (positive control, which was not treated) vs. RANKL (negative control, which was treated with only RANKL, ^###^ *p* < 0.001) and RANKL vs. sample treated group (*** *p <* 0.001).

To evaluate whether the AM extracts inhibited osteoclastogenesis, its effects on osteoclast differentiation in RANKL-induced RAW 264.7 cells were first investigated. The degree of osteoclast differentiation was indicated by the number of TRAP-stained cells according to TRAP staining analysis. The TRAP staining showed that all extracts inhibited the formation of mononuclear and multinuclear osteoclasts as the concentration of the extracts was increased (Figure 4a). However, AM ethanolic extract was found to be most potent among the extracts, resulting in decreased formation of TRAP-positive multinucleated osteoclasts, even at a concentration as low as 50 μg/mL. Furthermore, we investigated whether the extracts were effective on RANKL-stimulated TRAP activity in RAW 264.7 cells. The TRAP activity increased by up to 169% after treatment with RANKL compared with that of the control (Figure 4b). However, treatment with different concentrations of the extracts dose-dependently reduced the TRAP activity. Again, AM ethanolic extract was found to be the most effective of the extracts, which was well correlated with the analysis of TRAP staining. 

### 2.8. Aronia melanocarpa ‘Viking’ Extracts Inhibit MAP Kinase Activation in RANKL-Induced Osteoclasts

**Figure 5:**
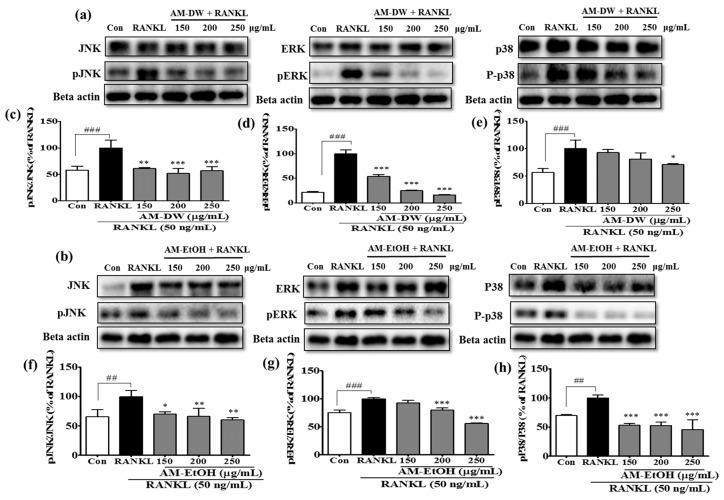
Inhibition of MAP kinase activation of RANKL induced osteoclast in RAW 264.7 cells by AM extracts. (**a**,**b**) AM water and ethanol extracts altered the phosphorylation of the MAPKs (JNK and ERK, p38); (**c**–**h**) Band intensities for pJNK/JNK, pERK/ERK, p-p38/p38 as percentages of the RANKL treated group (set as 100%), respectively for AM water and ethanol extracts. Cells were exposed to RANKL (50 ng/mL) in the presence and absence of AM extracts for 3 days. The expression of the proteins were determined by Western blot analysis and Beta actin was used as loading control. RANKL treated group was considered as 100% for densitometric analysis. All data are presented as the mean ± SD of three independent experiments performed with *n* = 3. * *p* < 0.05, ** *p* < 0.01, *** *p* < 0.001 vs. RANKL treated group; and ^##^ *p* < 0.01, ^###^ *p* < 0.001 vs. Con group. Con; positive control, (which was not treated), RANKL; negative control, (which was treated with only RANKL).

To gain further insights into the molecular mechanisms, intracellular signaling pathways were investigated to determine how the extracts suppressed osteoclast differentiation. MAPKs are crucial signaling molecules and during osteoclast differentiation, MAPKs signaling molecules transfer RANKL signals to the transcription factors in the nucleus from the cell surface receptor. ERKs, JNKs and p38, the three families of MAPKs, were reported to be activated by RANKL in osteoclast precursor cells [19]. We investigated the effects of the extracts on the phosphorylation level of ERK, JNK and p38 in RANKL-induced RAW 264.7 cells to identify whether MAPK signaling pathways were involved in the anti-osteoclastogenic function of the extracts. The phosphorylation of ERK, JNK and p38 was markedly inhibited by all the extracts (Figure 5). The AM ethanol extract was found to be more effective in the attenuation of p38 phosphorylation than the AM water extract. Interestingly, AM water extract was more potent to reduce the phosphorylation level of ERK as compared to AM ethanol extract. These findings suggested that the extracts affected the RANKL-induced MAPK signaling cascade, displaying a particularly prominent attenuation of the phosphorylation of JNK, ERK and p38.

### 2.9. Aronia melanocarpa ‘Viking’ Extracts Inhibit RANKL-Induced NF-κB Activation and Its Nuclear Translocation

**Figure 6:**
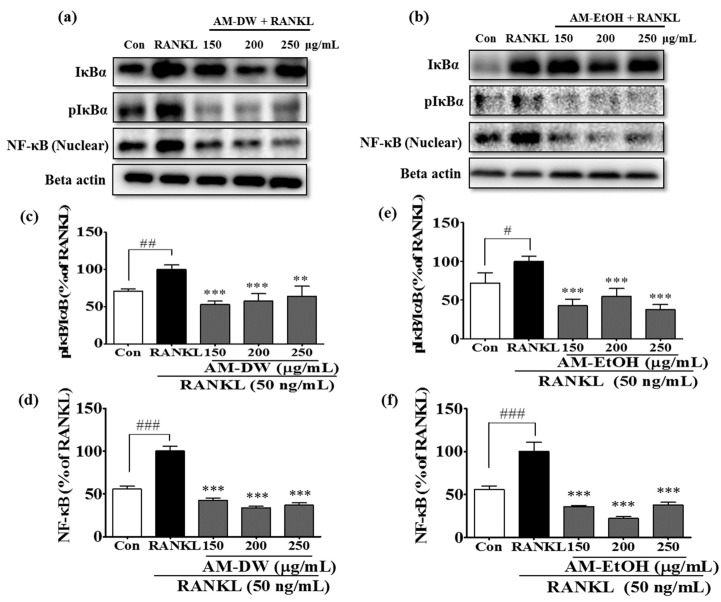
Inhibition of NF-κB activation of RANKL induced osteoclast in RAW 264.7 cells by AM extracts. (**a**,**b**) AM extracts altered the phosphorylation of the IκBα and inhibited nuclear translocation of NF-κB. (**c**–**f**) Band intensities of pIκBα/IκBα and NF-κB for AM water and ethanol extracts. Cells were exposed to RANKL (50 ng/mL) in the presence and absence of AM extracts for 3 days. The expression of the proteins were determined by Western blot analysis and Beta actin was used as loading control. RANKL treated group was considered as 100% for densitometric analysis. All data are presented as the mean ± SD of three independent experiments performed with *n* = 3. *** *p <* 0.001 vs. RANKL treated group; and ^#^ *p* < 0.05, ^##^ *p* < 0.01, ^###^ *p* < 0.001 vs. Con group. Con; positive control, (which was not treated), RANKL; negative control, (which was treated with only RANKL).

The activation of the transcription factor, NF-κB and its translocation into the nucleus is considered to be a crucial step in osteoclast differentiation. Recent studies suggested that NF-κB is an important upstream transcription factor that influences NFATc1 expression [20]. Therefore, we evaluated the phosphorylation of IκBα and the nuclear translocation of NF-κB. The phosphorylation of IκBα and the nuclear translocation of NF-κB were inhibited by the AM extracts (Figure 6).

### 2.10. Aronia melanocarpa ‘Viking’ Extracts Suppress Transcription Factor c-Fos and NFATc1 Expression in RANKL-Induced Osteoclasts 

**Figure 7:**
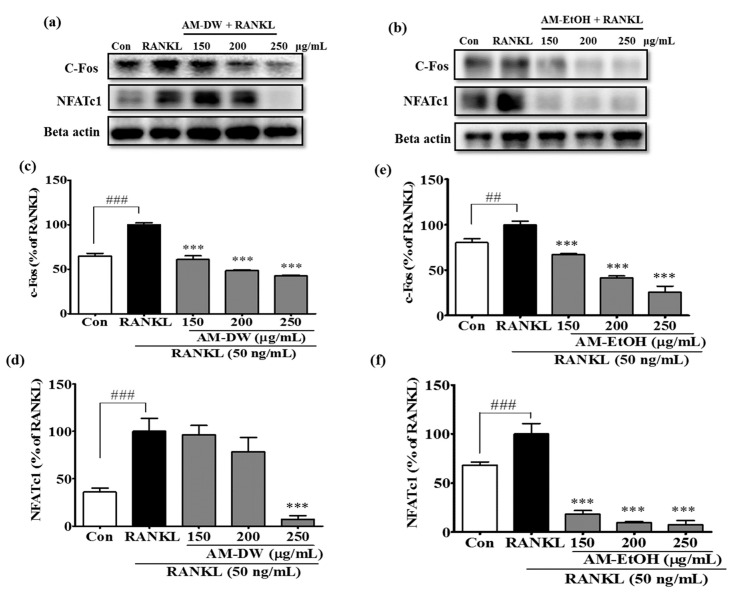
Inhibitory effects of AM extracts on c-Fos and NFATc1 expression in RAW 264.7 cells induced by RANKL. (**a**,**b**) AM extracts inhibited the expression of c-Fos and NFATc1; (**c**–**f**) Band intensities for c-Fos and NFATc1as percentages of the RANKL treated group (set as 100%), respectively for AM water and ethanol extracts. RANKL treated group was considered as 100% for densitometric analysis. Cells were exposed to RANKL (50 ng/mL) in the presence and absence of AM extracts for 3 days. The expression of the proteins were determined by Western blot analysis and Beta actin was used as loading control. All data are presented as the mean ± SD of three independent experiments performed with *n* = 3. *** *p <* 0.001 vs. RANKL treated group; and ^##^ *p* < 0.01, ^###^ *p* < 0.001 vs. Con group. Con; positive control, (which was not treated), RANKL; negative control, (which was treated with only RANKL).

Various transcription factors can be regulated by the translocation of activated MAPKs into the nucleus. c-Fos is an important transcription factor required for osteoclastic differentiation and activation by phosphorylated MAPKs, which is considered to participate in the induction of NFATc1. We evaluated whether the extracts influenced RANKL-induced c-Fos expression. RANKL stimulation clearly elevated c-Fos protein expression in RAW 264.7 cells. However, the presence of the extracts markedly lowered the upregulation of c-Fos protein expression (Figure 7).

NFATc-1 is a transcription factor considered to be a master gene for osteoclast differentiation and its expression can be induced by RANKL. Western blots were examined to determine the influence of the extracts on NFATc-1 protein expression. In response to RANKL, the expression of NFATc-1 was greatly upregulated. However, in the presence of the extracts the RANKL-dependent upregulation of NFATc-1 protein expression was substantially inhibited (Figure 7). These findings suggested that the suppression of the effects of the extracts was associated with the abolition of major transcription factors, such as c-Fos and NFATc-1.

### 2.11. Aronia melanocarpa ‘Viking’ Extracts Reduce Osteoclast Specific Gene Expression in RANKL-Induced RAW 264.7 Cells

**Figure 8:**
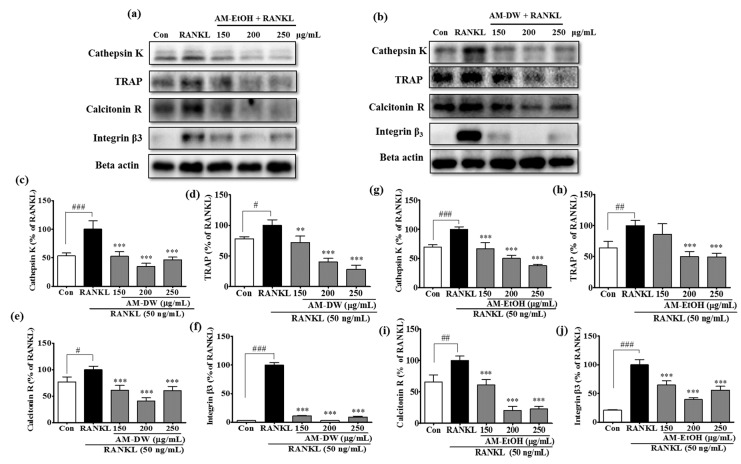
Inhibitory effects on gene expression of Cathepsin K, TRAP, Calcitonin receptor, Integrin β_3_ during RANKL induced osteoclast in RAW 264.7 cells by AM extracts. (**a**,**b**) AM extracts inhibited the expression of Cathepsin K, TRAP, Calcitonin receptor, Integrin β_3_; (**c**–**j**) Band intensities for Cathepsin K, TRAP, Calcitonin receptor, Integrin β_3_ as percentages of the RANKL treated group (set as 100%), respectively. RANKL treated group was considered as 100% for densitometric analysis. Cells were exposed to RANKL (50 ng/mL) in the presence and absence of AM extracts for 3 days. The protein expression was determined by Western blot analysis and Beta actin was used as loading control. All data are presented as the mean ± SD of three independent experiments performed with *n* = 3. ** *p* < 0.01, *** *p <* 0.001 vs. RANKL treated group; and ^#^ *p* < 0.05, ^##^ *p* < 0.01, ^###^ *p* < 0.001 vs. Con group. Con; positive control, (which was not treated), RANKL; negative control, (which was treated with only RANKL).

RANKL, through the activation of various signaling pathways, induces many osteoclast-specific genes, such as cathepsin K, TRAP, calcitonin receptor and integrin β_3_, which leads to increased osteoclast activity and bone resorption. The expression of osteoclast specific proteins has been investigated in RANKL-induced RAW 264.7 cells by using western blot. The protein expression of cathepsin K, TRAP, the calcitonin receptor and integrin β_3_ was significantly downregulated by AM extracts (Figure 8).

## 3. Discussion

ROS are continuously generated inside the body during general physiological events and lead to the initiation of membrane lipid peroxidation [21]. Studies have reported the positive effects of ROS, such as energy production, the regulation of cell growth and intracellular signaling. A wide range of essential cellular biomolecules, such as proteins, enzymes, DNA, RNA, lipids and carbohydrates are damaged by the overproduction of ROS [22]. Moreover, the activation of MAPK, down-stream signal depends on the ROS level. Antioxidants are reported to protect the human body from the action of free radicals and ROS [23]. Therefore, we evaluated the anti-oxidant capacity of AM extracts which can inhibit ROS production by scavenging free radical. The suppressive effect of AM extracts on ROS level can lead to less activation of MAPK and consequently inhibit the expression of cathepsin K, TRAP, integrin β_3_, calcitonin receptor by inhibiting JNK/ERK/p38, NF-κB-mediated c-Fos/NFATc1 signaling pathway. This study confirmed that AM extracts possessed strong radical scavenging activity that may play a crucial role in the inhibition of ROS generation. Moreover, Previous studies have reported that various flavonoid compounds, such as epigallocatechin-3-gallate [24], luteolin [25], baicalein [26] and fisetin [27], were associated with osteoclastic differentiation [28]. Many flavonoid and phenolic compounds, such as anthocyanin, A1 cyanidin 3-*O*-galactoside, flavonols, chlorogenic acid and F1 quercetin diglucoside, can be found in chokeberry [29]. We have identified four anthocyanin compounds by HPLC analysis such as cyanidin-3-galactoside, cyanidin-3-glucoside, cyanidin-3-arabinoside and cyanidin-3-xyloside. Therefore, these findings led us to examine the suppressive effects of AM extracts on RANKL-induced osteoclast differentiation through the ROS scavenging potential and its possible signaling pathways.

A low concentration of ROS-mediated signals is important for the activation of the downstream signaling pathway [30] and through the process of binding of various cytokines to receptors, the production of ROS is controlled [31]. During the osteoclast differentiation process, intracellular ROS generation is stimulated by the binding of RANKL to the RANK receptors. MAPKs, such as ERK, JNK and p38, are known to be ROS sensitive and responsible for the translocation of c-Fos and NFATc1 to the nucleus, which leads to the activation of cathepsin K and TRAP. Osteoclast differentiation is upregulated by this generation of ROS and participates in early signaling events that are related to osteoclast activation for bone resorption [32]. In this study, both AM extracts inhibited RANKL-induced ROS generation in RAW 264.7 cells. These results suggested that the extracts were able to downregulate MAPKs expression through the inhibition of ROS generation, which plays a crucial role as a secondary messenger in the RANKL osteoclast differentiation signaling pathway. In addition, it is noticed that, AM extracts did not able to inhibit ROS generation completely, therefore, we suggest that the inhibition of osteoclastogenesis by AM extracts was not solely dependent on ROS inhibition and it is also possible that, the inhibition of osteoclastogenesis by AM extracts occurred due to inhibition of the other signaling pathway. 

In mammalian cells, MAPKs consist of JNK, ERK and p38. In response to the extracellular response the MAPK group of enzymes phosphorylates serine and threonine residues and transmits the stimuli to the nucleus from the cell surface. An association between distinct signaling molecules, such as JNK, ERK and p38, is provided by the osteoclast precursor through the binding of RANKL to the RANK receptors. Several transcription factors, such as NF-κB, NFATc-1, c-Fos and AP-1, are induced by the activation of signaling molecules and these transcription factors are crucial for osteoclast differentiation. It has been reported that p38 upregulates the activity of microphthalmia-associated transcription factor (MITF) and TRAP expression. Therefore, p38 signaling is considered to be crucial in the initial stage of osteoclast differentiation [33,34]. In this study, AM extracts downregulated the phosphorylation of JNK, ERK and p38. Many transcription factors are considered to initiate the induction of genes implicated in osteoclastic differentiation induced by RANKL. Molecular genetic studies have revealed that NF-κB is essential for osteoclast differentiation. NF-κB can be activated by RANK through two signaling pathways, the canonical pathway involving the phosphorylation and subsequent degradation of IκB and the noncanonical pathway involving NIK-mediated p100 processing [35]. The IκB phosphorylation in the canonical NF-κB signaling pathway leads to its ubiquitination and proteasomal degradation, which consequently activates the translocation of NF-κB to the nucleus and influences transcriptional activity. The results of this study showed that AM extracts inhibited the processes of IκBα phosphorylation and nuclear translocation of NF-κB, both of which are associated with RANKL-induced osteoclastic differentiation. There are two key transcriptional factors, c-Fos and NFATc1, which are known to exert crucial roles in RANKL-induced osteoclastogenesis [36]. The binding of c-Fos to NFATc1 was induced by RANKL signaling, which consequently leads to the expression of NFATc1. Studies demonstrated that c-Fos was a major component of the AP-1 transcription factor complex, which includes members of the Jun family [37]. Osteoporosis developed in c-Fos deficient mice with an insufficiency in bone remodeling and tooth eruption [38]. Our study confirmed that AM extracts significantly downregulated the protein expression of RANKL-induced c-Fos and NFATc1. It is well established that NFATc1 is a downstream nuclear transcriptional factor and that it regulates the expression of various osteoclast-specific genes, such as cathepsin K, TRAP, integrin β_3_, c-Src and the calcitonin receptor. The organic bone matrix degraded by cathepsin K leads to bone resorptive activity. As cathepsin K and TRAP are expressed by osteoclasts, which are considered a functional biomarker in osteoclast differentiation, we investigated the protein expression of cathepsin K, TRAP, the calcitonin receptor and integrin β_3_. Our results confirmed that RANKL upregulated the expression of cathepsin K, TRAP, the calcitonin receptor and integrin β_3_ but treatment with the AM extracts significantly downregulated the protein expression. Therefore, AM extracts may inhibit osteoclastic differentiation through the suppression of the expression of these genes through the inactivation of c-Fos/NFATc1 signaling. 

A possible mechanism has been discussed here, how AM extracts affect osteoclast differentiation. When RANKL binds to the RANK receptor, the TNF receptor-associated factor (TRAF6) is activated and binding occurs in the cytoplasm [39]. It is believed that TRAF6 plays an important role in osteoclast differentiation among the TRAF protein family. Activated TRAF6 leads to the activation of NF-κB and MAPKs, such as ERK, JNK and p38. The transcription factor AP-1 is activated by JNK and c-Fos [40] and NF-κB with AP-1 regulate the early expression of NFATc1, which is a key transcription factor in osteoclast differentiation [41]. Through the process of auto-amplification, NFATc1 increases the transcription of NFATc1 and leads to the increased expression of osteoclast-specific genes, such as cathepsin k, TRAP, calcitonin R and integrin β_3_. The data suggested that AM extracts were able to inhibit ROS production and the activation of MAPKs and NF-κB, which leads to the downregulation of the expression of c-Fos and NFATc1 and consequently suppresses the expression of osteoclast-specific genes.

Collectively, the intracellular ROS can be controlled through the generation and scavenging of ROS. AM extracts were found to be rich in phenolic and flavonoid contents and exhibited strong free radical scavenging activity, by which AM extracts are able to inhibit excess accumulation of ROS. Moreover, our study confirmed that the extracts inhibited the production of ROS stimulated by RANKL in RAW 264.7 cells. It could be possible that, the suppressive effects of AM extracts on ROS level leads to the less activation of MAPK and eventually inhibit the expression of TRAP, cathepsin K, integrin β_3_, calcitonin receptor by blocking c-Fos and NFATc1 up-stream signaling pathway. The extracts were also found to inhibit TRAP-positive osteoclast formation. In addition, this study revealed the underlying molecular mechanism and demonstrated that the AM extracts inhibited osteoclastogenesis through inactivation of MAPKs, which leads to the down regulation of c-Fos and NFATc1 up-stream signaling and suppress the protein expression of osteoclast specific genes induced by RANKL in RAW 264.7 cells. It is suggested that, AM extracts possess anti-osteoclastic activity owing to the high content of phenolic compounds, especially anthocyanin. In conclusion, AM extracts, or their derivatives may be potentially useful therapeutic agents for the prevention or treatment of bone loss-associated diseases. 

## 4. Materials and Methods

### 4.1. Chemicals and Materials

Folin-ciocalteau (FC reagent), gallic acid, 2,2-azino-bis (3-ethylbenzo thiazoline-6-sulfonic acid) diammonium salt (ABTS), sodium nitrite, sulfanilic acid, acetic acid, sodium citrate, ascorbic acid and ferrous sulfate heptahydrate (FeSO_4_) were obtained from Sigma-Aldrich Co. LLC (St. Louis, MO, USA). 1,1-Diphenyl-2-picrylhydrazyl (DPPH) was obtained from Wako Pure Chemical Industries Ltd. (Osaka, Japan). D-Catechin was purchased from MP Biomedicals, LLC (Illkich, France). Hydrogen peroxide 30% was obtained from Daejung Chemicals and Metals Co. (Gyeonggi-do, Korea). pBR322 DNA and 6× DNA loading dye were purchased from Thermo Fisher Scientific Inc. (NYSE: TMO, Waltham, MA USA; Gyeonggi-do, Korea). RAW 264.7 cells were purchased from the Korean Cell Line Bank (Seoul, Korea). DMEM, penicillin-streptomycin and fetal bovine serum (FBS) were purchased from GIBCO BRL (Life Technologies, Grand Island, NY, USA). All chemicals were used without any further purification.

### 4.2. Preparation of Extracts

*Aronia melanocarpa* ‘Viking’ fruit freeze dried powder was immersed in distilled water/70% ethanol and maintained at room temperature for 24 h. Subsequently, the extracts were passed through Whatman No. 1 filter paper. This process was repeated three times. After collection, the extracts were evaporated under reduced pressure at 50 °C in a vacuum rotary evaporator (Eyela, Tokyo, Japan). The concentrated mass was freeze-dried, weighed and stored in a refrigerator at −20 °C until use. The yields were calculated from the following equation: Y (%) = (total extracted sample weight/total dry sample weight) × 100. *Aronia melanocarpa* ‘Viking’ fruit dried powder was obtained from Integrated Agricultural products Processing Center (Gangwon-do, Jeongseon-gun, Song Seok-gil, Korea). The plant material was identified by the ‘Korea Seed and variety Service’ (KSVS) and the sample (ARONIA600) has been deposited at the Bio Food and Drug Material Laboratory, Department of applied Life Science, Konkuk University, South Korea. 

### 4.3. High-Performance Liquid Chromatography (HPLC) Analysis 

Anthocyanin was analyzed using Thermo Scientific Dionex Ultimate 3000 Series, equipped with syncronis C18, 5 μm, 250 mm × 4.6 mm column (Thermo Fisher Scientific, Walthan, MA, USA). To identify the anthocyanins, a 0.1% water solution of TFA was used as solvent A and HPLC-grade acetonitrile was used as solvent B (elution conditions: 0–5 min, 5–10% B; 5–10 min, 15% B; 10–15 min, 18% B; 15–20 min, 20% B; 20–25 min, 25% B; 25–35 min, 30% B; flow rate, 1 mL/min; injection volume, 10 μL; wavelength, 520 nm visible and column temperature 40 °C). Prior to injection into the HPLC system, the extracts were diluted with methanol and passed through a 0.45 μm membrane filter. The data was collected by CHROMELEON Chromatography Management System (Version: 6.80). Standard curves were made using external standards such as cyaniding-3-galactoside, cyaniding-3-glucoside, cyaniding-3-arabinoside, cyaniding-3-xyloside for quantification. Cyanidin-3-galactoside, cyanidin-3-glucoside were obtained from Sigma-Aldrich (St. Louis, MO, USA); cyaniding-3-arabinoside was purchased from Chem Faces (Wuhan, China) and cyaniding-3-xyloside purchased from Toronto Research Chemicals (Toronto, ON, Canada). The identification was confirmed by UV-VIS spectrophotometer (wavelength 520 nm), comparing with those of standards.

### 4.4. Determination of Total Phenolic Content

The total amount of phenolic content was determined by the Folin-Ciocalteu method [42] with gallic acid used as a standard. At first, samples were prepared (10 mg/mL) in water and ethanol and different concentrations of gallic acid (5–100 μg/mL) were prepared in water. Next, 40 μL of sample solution, 20 μL of 1 N FC reagent and 60 μL of sodium carbonate (20%, *w*/*v*) were mixed. To complete the reaction, the mixture was stored at room temperature for 30 min in the dark. Finally, the absorbance was measured at 700 nm by using a UV-visible spectrophotometer (Sunrise Basic Tecan, Grödig, Austria). A standard calibration curve of gallic acid was constructed to measure the total phenolic content. The results were expressed as mg gallic acid equivalents (GAE) per 100 g of dry mass.

### 4.5. Determination of Total Flavonoid Content

The total flavonoid contents were measured by the aluminum colorimetric method used by Sakanaka and colleagues [43]. The extracted samples were dissolved in water and ethanol at 10 mg/mL and the concentration of catechin, which was used as the standard, was between 2.5 and 25 μg/mL. Initially, 25 μL of each sample/standard solution was mixed with 125 μL DW and 8 μL of 5% sodium nitrite solution and incubated for 5 min. Next, 15 μL of 10% *w*/*v* aluminum chloride solution was added to the mixture and incubated for 6 min at room temperature. Subsequently, 50 μL of 1 M sodium hydroxide and 27 μL distilled water were added. Finally, the absorbance was measured at 510 nm by using a UV spectrophotometer.

### 4.6. DPPH Radical Scavenging Activity

The DPPH radical scavenging activities of the extracts were analyzed in accordance with the method of Lee et al. [44]. In detail, different concentrations (50–200 μg/mL) of the extracts (dissolved in water and ethanol) and ascorbic acid (positive control) were dissolved in water. Then, 80 μL of 0.2 mM DPPH solution was added to 80 μL of each sample/standard solution. After that, the mixture was shaken for 30 min in the dark at room temperature. Finally, the absorbance was measured at 517 nm. The DPPH radical scavenging activity was calculated from the following equation:

DPPH radical scavenging activity (%) = {(C − D) − (A − B)/(C − D)} × 100(1)

where A is the absorbance of the DPPH + sample/standard, B is the absorbance of the sample/standard + methanol, C is the absorbance of DPPH + distilled water/ethanol and D is the absorbance of methanol + distilled water/ethanol.

### 4.7. ABTS Radical Scavenging Activity

ABTS radical scavenging activity was measured following a previously described method [45] with slight modifications. ABTS was diluted in distilled water at a concentration of 7 mM and mixed with 2.45 mM potassium persulfate. The mixture was then kept at room temperature for 12–16 h in the dark. Freshly prepared ABTS+ solution was diluted with 0.1 M phosphate-buffered saline (PBS, pH 7.4) to adjust its absorbance at 734 nm to within 0.70 ± 0.02. Subsequently, 0.8 mL ABTS+ solution was mixed with different concentrations (50–200 μg/mL) of the sample and ascorbic acid (used as the standard) and left for 5 min to complete the reaction. The scavenging activity of ABTS free radical was calculated from the following equation:

ABTS scavenging activity (%) = {(C − D) − (A − B)/(C − D)} × 100(2)

where A is the absorbance of ABTS solution + sample/standard, B is the absorbance of potassium persulfate + sample/standard, C is the absorbance of ABTS solution + distilled water/ethanol and D is the absorbance of potassium persulfate + distilled water/ethanol.

### 4.8. Determination of Reducing Power

The reducing power ability was determined by a previously reported method [46] method with minor modification. First, 0.5 mL of each sample and standard (50–200 μg/mL) was mixed with 0.5 mL of 0.2 M sodium phosphate buffer (pH 6.6) and 0.5 mL potassium ferricyanide (10 mg/mL). The mixture was then incubated in a water bath for at 40 °C for 20 min. After incubation, 1 mL of each mixture was isolated and added to 0.5 mL of 10% TCA and centrifuged at 3000 rpm for 10 min at 4 °C. Next, 0.5 mL of the supernatant was separated and mixed with 0.5 mL distilled water and 0.1 mL of 0.1% *w*/*v* FeCl_3_. The absorbance was measured at 7 nm against a standard of ascorbic acid.

### 4.9. Cell Culture

The murine macrophage RAW 264.7 cell was purchased from the Korean Cell Line Bank (Seoul, Korea) and cultured in DMEM medium supplemented with 10% heat-inactivated fetal bovine serum, penicillin (100 units/mL) and streptomycin (100 μg/mL). The cells were stored in a humid atmosphere containing 5% CO_2_ at 37 °C. 

### 4.10. Cell Viability Analysis

Cell viability was determined by using an MTT assay. RAW 264.7 cells (5 × 10^4^ cells/well) were seeded in 96-well plates. After incubation for 24 h at 37 °C, the cells were treated with different concentrations of the extracts in the presence of RANKL and without RANKL. Next, the MTT solution (2 mg/mL) was added in each well and the plates were incubated in the dark at 37 °C. The culture medium was removed and DMSO was added. The absorbance was measured at 540 nm by using a microplate absorbance reader (Tecan Austria GmbH, Salzburg, Austria).

### 4.11. Determination of Intracellular Reactive Oxygen Species (ROS)

The generation of intracellular ROS was evaluated through the measurement of the oxidation of 2′,7′-dichlorodihydrofluorescein diacetate (H_2_DCF-DA) to fluorescent 2′,7′-dichlorofluorescein (DCF) by hydroperoxides. At first, RAW 264.7 cells (5 × 10^4^ cells/well) were seeded in 96-well plates. After incubation for 24 h, the cells were treated with different concentrations of the sample (10–500 μg/mL) for 24 h. Subsequently, 10 μM 2′,7′-dichlorodihydrofluorescein diacetate (H_2_DCF-DA) was added to the cell, incubated for 40 min, 50 μM H_2_O_2_ or 50 ng/mL RANKL was then added and the fluorescence was detected after 1 h. The fluorescence was measured by using a Soft Max Pro 5 at the excitation and emission wavelengths of 485 and 530 nm, respectively.

### 4.12. Tartrate-Resistant Acid Phosphatase (TRAP) Activity

RAW 264.7 cells were seeded in 96-well plates (5 × 10^3^ cells/well) with DMEM medium supplemented with 10% FBS and 1% penicillin-streptomycin. After incubation for 24 h, the medium was replaced by test samples with differentiation medium (α-MEM) containing 50 ng/mL RANKL. After 3 days, the cells were washed with PBS and 80 μL lysis buffer (90 mM citrate, pH 4.8, 0.1% Triton X-100 containing 80 mM sodium tartrate) was added and incubated for 10 min. Subsequently, 80 μL of the substrate solution (20 mM *p*-nitrophenyl phosphate) was added to the mixture and incubated for 20 min at 37 °C in an atmosphere of 5% CO_2_. Next, 40 μL of 0.5 N NaOH was added. The absorbance was measured at 405 nm wavelength by using a UV spectrophotometer (TECAN AUSTRIA GMBH, Salzburg, Austria).

### 4.13. Tartrate-Resistant Acid Phosphatase (TRAP) Staining

After incubation for 5 days with differentiation media containing sample/RANKL, the cells were fixed with 4% paraformaldehyde for 5 min and washed with warm distilled water. In accordance with the instructions of the Sigma TRAP staining kit (Kit No. 387; Sigma-Aldrich, St. Louis, MO, USA), freshly prepared TRAP solution was added for 30 min. A microscope (CKX41, Olympus, Tokyo, Japan) was used to visualize the TRAP-positive osteoclasts and the images were captured by using a digital video camera (eXcope T500, Olympus, Tokyo, Japan).

### 4.14. Preparation of Total Cell Extracts and Immunoblot Analysis 

RAW 264.7 cells (8 × 10^3^ cells/well) were seeded in 6-well plates with DMEM culture medium supplemented 10% FBS and 1% penicillin-streptomycin and incubated for 24 h at 37 °C in an atmosphere of 5% CO_2_. After incubation, the cells were treated by 50 ng/mL RANKL and extracts with α-MEM (containing 10% FBS and 1% penicillin-streptomycin). After 3 days, the whole-cell extract was collected by the addition of PRO-PREP buffer (iNtRON Biotechnology). The nuclear protein was collected by using NE-PER nuclear and cytoplasmic extraction reagents according to the manufacturer’s instructions (Thermo Scientific). A BSA protein assay was used to determine the protein concentration of all the extracts. The extracts were subjected to SDS-polyacrylamide gel electrophoresis and transferred to a nitrocellulose membrane. The membranes were blocked with 5% BSA, probed with primary antibodies and incubated at 4 °C overnight. Horseradish peroxidase-conjugated secondary antibodies were then added. The targeted proteins were visualized by using enhanced chemiluminescence. The images were obtained by using the BIO-RAD ChemiDoc XRS+ (BIO-RAD, Philadelphia, PA, USA) imaging system and Image Lab software.

### 4.15. Statistical Analysis

All data are presented as the mean ± standard deviation from three independent experiments. All statistical analyses were computed by using GraphPad Prism 5.0 software (GraphPad Software, Inc., San Diego, CA, USA). The significance of each group was determined by using one-way analysis of variance (ANOVA) followed by Dunnett’s multiple comparison test or Bonferroni post-tests. A value of *p* < 0.05 was considered to represent a statistically significant difference.

## Figures and Tables

**Table 1:** Content of anthocyanin compounds of *Aronia melanocarpa* ‘Viking’ extracts.

Peak	Compound	Content (mg/g)	
		AM-DW	AM-EtOH
**1**	Cyanidin-3-galactoside	4.7131	9.9636
**2**	Cyanidin-3-glucoside	0.4560	0.9497
**3**	Cyanidin-3-arabinoside	1.2700	3.5179
**4**	Cyanidin-3-xyloside	0.1860	1.1423

**Table 2:** Total phenolic and flavonoid content and yield of *Aronia melanocarpa* ‘Viking’.

Extract	Total Phenolic (mg GAE ^†^/100 g of Dry Mass)	Total Flavonoid (mg CE ^‡^/100 g of Dry Mass)	Yield (%)
AM-DW	2617.25 ± 14.96	2004.99 ± 26.87	62.85
AM-EtOH	4004.46 ± 49.21	3896.11 ± 13.74	64.30

All data are expresses as mean ± standard deviation (*n* = 3). ^†^ GAE: gallic acid equivalent; ^‡^ CE: catechin equivalent.
